# Macrophage‐targeted delivery of siRNA to silence *Mecp2* gene expression attenuates pulmonary fibrosis

**DOI:** 10.1002/btm2.10280

**Published:** 2022-01-18

**Authors:** Yong Mou, Guo‐Rao Wu, Qi Wang, Ting Pan, Lei Zhang, Yongjian Xu, Weining Xiong, Qing Zhou, Yi Wang

**Affiliations:** ^1^ Department of Respiratory and Critical Care Medicine, NHC Key Laboratory of Pulmonary Diseases, Key Site of National Clinical Research Center for Respiratory Disease, Wuhan Clinical Medical Research Center for Chronic Airway Diseases, Tongji Hospital, Tongji Medical College Huazhong University of Science and Technology Wuhan China; ^2^ Department of Pulmonary and Critical Care Medicine, The Central Hospital of Wuhan, Tongji Medical College Huazhong University of Science and Technology Wuhan China; ^3^ Department of Respiratory and Critical Care Medicine, Shanghai Key Laboratory of Tissue Engineering, Shanghai Ninth People's Hospital Shanghai Jiaotong University School of Medicine Shanghai China; ^4^ The Center for Biomedical Research, Tongji Hospital, Tongji Medical College Huazhong University of Science and Technology Wuhan China

**Keywords:** alternatively activated macrophages, idiopathic pulmonary fibrosis, liposomes, macrophages, Mecp2

## Abstract

Idiopathic pulmonary fibrosis (IPF) is a progressive interstitial lung disease characterized by the infiltration of macrophages in the fibrotic region. Currently, no therapeutic strategies effectively control disease progression, and the 5‐year mortality of patients after diagnosis is unacceptably high. Thus, developing an effective and safe treatment for IPF is urgently needed. The present study illustrated that methyl‐CpG‐binding protein 2 (MECP2), a protein responsible for the interpretation of DNA methylome‐encoded information, was abnormally expressed in lung and bronchoalveolar lavage fluid samples of IPF patients and mice with onset of pulmonary fibrosis. And further studies verified that the overexpression of MECP2 occurred mainly in macrophages. Inhibition of Mecp2 expression in macrophages robustly abrogated alternatively activated macrophage (M2) polarization by regulating interferon regulatory factor 4 expression. Accordingly, cationic liposomes loading *Mecp2* small interfering RNA (siRNA) were raised for the treatment of pulmonary fibrosis. It was noted that the liposomes accumulated in the fibrotic region after intratracheal injection, especially in macrophages. In addition, intratracheal administration of *Mecp2* siRNA‐loaded liposomes significantly reversed the established pulmonary fibrosis with few side‐effects and high safety coefficients. Collectively, these results are essential not only for further understanding the DNA methylation in pathogenesis of IPF but also for providing a potent therapeutic strategy for IPF treatment in the clinic practice.

## 1 INTRODUCTION

Idiopathic pulmonary fibrosis (IPF) is a progressive, lethal fibrotic lung disease with unknown etiology.^1^ Although pirfenidone and nintedanib are approved by the FDA and modify disease progression in some patients, IPF still carries a poor prognosis, with a median survival of 3.8 years among adults 65 years of age or older.^1^ Therefore, it is necessary to develop safe and effective therapeutic strategies for IPF that can be used in clinical practice.

Macrophages are the most abundant immune cells in normal lungs (~70% of immune cells) and are characterized by their plasticity.^2^ These cells are activated by Th1 cytokines and/or microbial agents to exhibit a classically activated phenotype (M1) or by Th2 cytokines to exhibit an alternatively activated phenotype (M2).^3^ A growing body of evidence supports that macrophages actively participate in the pathogenesis of IPF.^2^, ^4^ Specifically, these cells produce a myriad of profibrotic mediators, such as transforming growth factor‐beta 1 (TGF‐β1), platelet‐derived growth factor (PDGF), and found in inflammatory zone 1 (Fizz1), to promote fibroblast differentiation, proliferation and migration, leading to high levels of extracellular matrix (ECM) deposition in the lung parenchyma, impaired functional gas exchange, respiratory failure, and even death.^2^, ^5^, ^6^ Indeed, modulating M2 macrophage polarization is a feasible strategy for the treatment of pulmonary fibrosis.^7^

DNA methylation is a bridge between environmental stimuli and gene expression. Recent studies have revealed that DNA methylation is involved in the pathogenesis of IPF.^8^, ^9^ Methyl‐CpG‐binding protein 2 (Mecp2), a member of the methyl‐CpG‐binding domain (MBD) protein family, is responsible for the interpretation of DNA methylome‐encoded information.^10^ In general, Mecp2 directly binds to methylated CpG DNA and then recruits other remodelers or enzymes to form a complex, leading to the repression or activation of gene expression.^11^ There is weak evidence that Mecp2 is involved in the pathogenesis of pulmonary fibrosis.^12^ However, the exact mechanism has yet to be fully elucidated. Our previous study illustrated that DNA methylation participates in M2 macrophage polarization.^13^ Interestingly, aberrant expression of MECP2 was detected in the lung and bronchoalveolar lavage fluid (BALF) samples of IPF patients and mice with pulmonary fibrosis, and this aberrant expression occurred primarily in macrophages. These observations prompted us to hypothesize that MECP2 might orchestrate M2 macrophages polarization during the development of IPF. Herein, we provided convincing evidence that silencing *Mecp2* expression alleviated the M2 program by regulating interferon regulatory factor 4 (Irf4) expression.

RNA interference (RNAi)‐based therapy has emerged as a promising therapeutic strategy in chronic diseases due to its excellent ability to silence gene expression in a highly sequence‐specific manner.^14^ In general, RNAi delivers specific small interfering RNA (siRNA) to target tissues and/(or) cells with fewer side‐effects and more effectiveness than traditional therapies.^15^ In the current study, we showed that *Mecp2* siRNA‐loaded liposomes specifically targeted macrophages and passively accumulated in the pulmonary fibrotic areas of mice with pulmonary fibrosis following intratracheal injection. Notably, treatment with *Mecp2* siRNA‐loaded liposomes robustly reversed established pulmonary fibrosis in a macrophage dependent manner. Collectively, our data suggest that Mecp2 is essential for the progression of pulmonary fibrosis, and therefore, intratracheal injection of *Mecp2* siRNA‐loaded liposomes could be a viable therapeutic approach for pulmonary fibrosis.

## 2 MATERIALS AND METHODS

### 2.1 Reagents and antibodies

Bleomycin (BLM) was obtained from Huirui. Murine recombinant IL‐4 was purchased from PeproTech. The Lipofectamine 3000 transfection kit was acquired from Invitrogen. Cholesterol and DSPC were purchased from Sigma‐Aldrich, Inc. Lipidoid (C12‐200) was acquired from Xinjiahecheng Medical Chemistry Corporation. mPEG2000‐DEG was purchased from NOF Corporation. siRNAs targeting *Mecp2* and Scr siRNA were purchased from Guangzhou RiboBio Co., Ltd.

Antibodies against CD68, CD206, and TGF‐β1 were purchased from Santa Cruz Biotechnology. Antibodies against arginase‐1 and fibronectin were purchased from Abcam. Antibodies against Mecp2, IRF4, inducible nitric oxide synthase (iNOS), and α‐SMA were purchased from Cell Signaling Technology. Antibodies against collagen I, Irf4, Gapdh, and β‐actin were purchased from Proteintech, and antibodies against Ym1 were purchased from Thermo Fisher Scientific. APC‐conjugated anti‐mouse F4/80, PE‐conjugated anti‐mouse CD11c, and FITC‐conjugated anti‐mouse CD206 antibodies were purchased from BioLegend.

### 2.2 Human samples

Human lung tissues were collected from patients with non‐small cell lung cancer (*n* = 5) and IPF (*n* = 5). BALF was collected from healthy volunteers and IPF patients at Tongji Hospital, followed by informed consent. The diagnosis of IPF was made according to the American Thoracic Society/European Respiratory Society consensus diagnostic criteria.^16^ The Human Assurance Committee of Tongji Hospital approved all clinical studies (IRB: TJ‐IRB20210942). The clinical features and pulmonary functional analyses of patients were shown in Table 1.

**TABLE 1:** Characteristics of subjects for lung samples

	Lung samples	
	IPF (*n* = 5)	Control (*n* = 5)
Age (years)	61.80 ± 12.22	57.45 ± 8.55
BMI	22.82 ± 3.36	22.89 ± 3.14
Sex		
Female	2 (40.00%)	2 (40.00%)
Male	3 (60.00%)	3 (60.00%)
FVC		
Percent predicted	74.09 ± 15.96	NA
DLCO	46.34 ± 5.49	NA

Abbreviations: BMI, body mass index; DLCO, diffusion capacity for carbon monoxide; FVC, forced vital capacity.

### 2.3 Animals studies

Eight‐week‐old male C57BL/6 mice were obtained from Beijing Vital River Laboratory Animal Technology Co., Ltd. The mice were maintained under specific pathogen‐free conditions. Induction of pulmonary fibrosis in C57BL/6 mice was performed by intratracheal injection of BLM (2 U/kg) or phosphate‐buffered saline (PBS; as a control) with a high pressure atomizing needle (Cat: BJ‐PW‐M; Bio Jane Trading Limited) after anesthetizing with pentobarbital sodium (60 mg/kg). For the therapeutic experiments, the mice were administered *Mecp2* siRNA‐loaded liposomes, scramble (Scr) siRNA‐loaded liposomes or liposomes (1 mg/kg) via intratracheal injection on Days 14 and 17 after BLM induction, as shown in Figure 5a. The mice were sacrificed 21 days after BLM induction and lung fibrosis was analyzed.

In the macrophage depletion experiments, mice were divided into two groups: (I) BLM + clodronate liposomes + Scr siRNA‐loaded liposomes group; (II) BLM + clodronate liposomes + *Mecp2* siRNA‐loaded liposomes group. Clodronate liposomes (15 mg/kg) were administered intratracheally on Day 8 after BLM treatment. The Scr siRNA‐loaded liposomes and *Mecp2* siRNA‐loaded liposomes were administered intratracheally on Days 10 and 14 after BLM treatment. Finally, the mice were analyzed on Day 17 after BLM induction (Figure 7a).

All animal protocols and procedures were performed according to NIH guidelines and were approved by the Animal Care and Use Committee of Tongji Hospital (IRB: TJH‐201901015).

### 2.4 Western blotting analysis

Mouse and human lung tissues were homogenized in RIPA lysis buffer (Beyotime) containing a protease inhibitor cocktail (Roche), and equal amounts of lysates were separated on 10% polyacrylamide gels (Sigma‐Aldrich) and transferred onto polyvinylidene difluoride membranes as previously described.^17^ Target protein analysis was performed as described using appropriate primary antibodies, followed by probing with the corresponding horseradish peroxidase‐conjugated secondary antibodies. The reactive bands were visualized using ECL reagents (Servicebio), and the band intensities were analyzed using ImageJ software.

### 2.5 Histological and immunofluorescence staining

Human lung tissue and the left lung of mice were inflated in fresh 4% paraformaldehyde for 24 h at room temperature. Then, the lung tissue was embedded in paraffin and sliced into 5 μm sections. The sections were subjected to hematoxylin and eosin (H&E), Sirius red and Masson's trichrome staining. The Ashcroft scores of the mice were determined to assess the severity of lung fibrosis in each mouse by a blinded observer according to the established protocol.^18^ For immunofluorescence staining, BALF cytospin slides or paraffin sections were probed with antibodies against CD68, MECP2, and IRF4, followed by staining with Alexa Fluor 594–labeled anti‐mouse/rabbit or Alexa Fluor 488–conjugated anti‐rabbit/mouse antibodies (Invitrogen).

### 2.6 Cell culture

Primary bone marrow‐derived macrophages (BMDMs) were isolated and differentiated with M‐CSF as previously described.^19^ BMDMs were cultured in Roswell Park Memorial Institute‐1640 medium (Gibco) with 10% fetal calf serum and antibiotics (penicillin/streptomycin) (Beyotime). BMDMs were treated with 10 ng/ml IL‐4 after 7 days of M‐CSF‐mediated differentiation. For the siRNA transfection experiments, BMDMs were transfected with *Mecp2* siRNA (100 nM) or Scr siRNA (100 nM) on Day 5, and IL‐4 or LPS stimulation was performed on Day 7. The cells were then harvested for Western blotting, reverse transcription‐polymerase chain reaction (RT‐PCR), and flow cytometry.

### 2.7 siRNA transfection

BMDMs were transfected with *Mecp2* siRNA or Scr siRNA by using Lipofectamine 3000 reagent (Invitrogen) on Day 5 of M‐CSF differentiation according to the manufacturer's instructions. Forty‐eight hours after transfection, the cells were stimulated with murine IL‐4 (10 ng/ml) or LPS (100 ng/ml) for the indicated times.

### 2.8 Flow cytometry

*Mecp2* or Scr siRNA‐transfected BMDMs were digested after IL‐4 stimulation for 24 h and incubated with staining buffer containing APC‐conjugated anti‐mouse F4/80 and FITC‐conjugated anti‐mouse CD206 antibodies at 4°C for 30 min, after which the samples were washed twice with PBS prior to flow cytometric analysis. *Mecp2* or Scr siRNA‐treated mice were sacrificed and the lung tissues were collected and digested. After digestion, cells were stained by APC‐conjugated anti‐mouse F4/80, PE‐conjugated anti‐mouse CD11c and FITC‐conjugated anti‐mouse CD206 antibodies. Flow cytometric analysis of liposomes distribution was conducted as follows. On Day 19 after BLM induction, the mice were administered DiO‐labeled liposomes via intratracheal injection and sacrificed on Day 21. The resuspended cells were stained with staining buffer containing APC‐conjugated anti‐mouse F4/80 at 4°C for 30 min. Finally, the samples were washed twice with PBS prior to flow cytometric analysis. Data were acquired on a MACSQuant X (Miltenyi) and analyzed using FlowJo V10 software.

### 2.9 RT‐PCR

Total RNA was isolated from the mouse lung and cultured cells with the TRlzol reagent (Takara). The RNA quantity and quality were measured using a NanoDrop 2000 spectrophotometer (Thermo Fisher Scientific). cDNA was prepared using a cDNA synthesis kit (Takara). Quantitative RT‐PCR analysis was performed using SYBR Premix Ex Taq (Takara) as previously described.^20^ The primer sequences for the target genes are listed in Table 2. All reactions were performed in triplicate.

**TABLE 2:** Primers for RT‐PCR

Gene	Sequence (5′–3′)
*Fn1*‐F	TCTGGGAAATGGAAAAGGGGAATGG
*Fn1*‐R	CACTGAAGCAGGTTTCCTCGGTTGT
*Coll1α*‐F	GGAGGGCGAGTGCTGTGCTTT
*Coll1α*‐R	GGGACCAGGAGGACCAGGAAGT
*Acta2*‐F	GGCTGTATTCCCCTCCATCG
*Acta2*‐R	CCAGTTGGTAACAATGCCATGT
*Mecp2*‐F	ATGGTAGCTGGGATGTTAGGG
*Mecp2*‐R	TGAGCTTTCTGATGTTTCTGCTT
*Irf4*‐F	TCCGACAGTGGTTGATCGAC
*Irf4*‐R	CCTCACGATTGTAGTCCTGCTT
*Gapdh*‐F	AGGTCGGTGTGAACGGATTTG
*Gapdh*‐R	TGTAGACCATGTAGTTGAGGTCA

Abbreviation: RT‐PCR, reverse transcription‐polymerase chain reaction.

### 2.10 Preparation of siRNA‐loaded liposomes

siRNA‐loaded liposomes were constructed as previous described.^6^, ^21^ Briefly, the lipid composition was lipidoid (C12‐200): cholesterol: distearoyl phosphatidylcholine (DSPC): 1,2‐dimyristoyl‐rac‐glycero‐3‐methoxypolyethylene glycol‐2000 (mPEG‐DMG) dissolved in ethanol (50:38.5:10:1.5 molar ratio). siRNA was dissolved in citrated buffer (10 mM, pH 3). Then, the lipid components and dissolved siRNA were mixed rapidly by vortex. And ultrafiltration centrifugation was used to exclude unentrapped siRNA. Finally, the prepared siRNA‐liposomes were diluted in PBS at 4°C. The liposomes characteristics (hydrodynamic diameter, zeta potential, polydispersity and stability) listed in Figure S3 were measured by dynamic light scattering (Malvern Zetasizer Nano‐ZS). The entrapment efficiency and loading efficiency of the liposome were calculated by a Ribogreen assay. After staining with 2% phosphotungstic acid, the liposomes morphology was observed by transmission electron microscopy (TEM; JEM‐1230).

### 2.11 Hydroxyproline level analysis

The lung hydroxyproline level was measured with a hydroxyproline assay kit from Nanjing Jiancheng Institute of Biotechnology as previously described.^22^ Briefly, the fresh lung tissues were weighed and alkaline hydrolyzed for 20 min at 100°C. After adjusting pH to 6.0–6.8, the hydrolysates were refined with active carbon and centrifuged at 3500 rpm for 10 min. The supernatants were then undergone a series of chemical reactions, and finally OD values were determined at 550 nm using a microplate reader (ELx800; BioTek Instruments, Inc.). The hydroxyproline content in the lung tissue was given as μg hydroxyproline per mg lung tissue by comparing with the hydroxyproline standard.

### 2.12 Statistical analysis

Comparisons between groups were undertaken using the Prism software (GraphPad Prism 8.lnk; GraphPad Software, Inc). Two experimental groups were compared using the two‐tailed Student's *t*‐test or two‐tailed Mann–Whitney test. Once more than two groups were compared, one‐way or two‐way analysis of variance with Tukey's multiple comparison test or Kruskal–Wallis test with Dunn's posthoc tests were used. The data are presented as the mean ± *SEM*. In all cases, *p* < 0.05 was considered with statistical significance.

## 3 RESULTS

### 3.1 MECP2 was specifically expressed in macrophages within the fibrotic lungs of IPF patients

**FIGURE 1:**
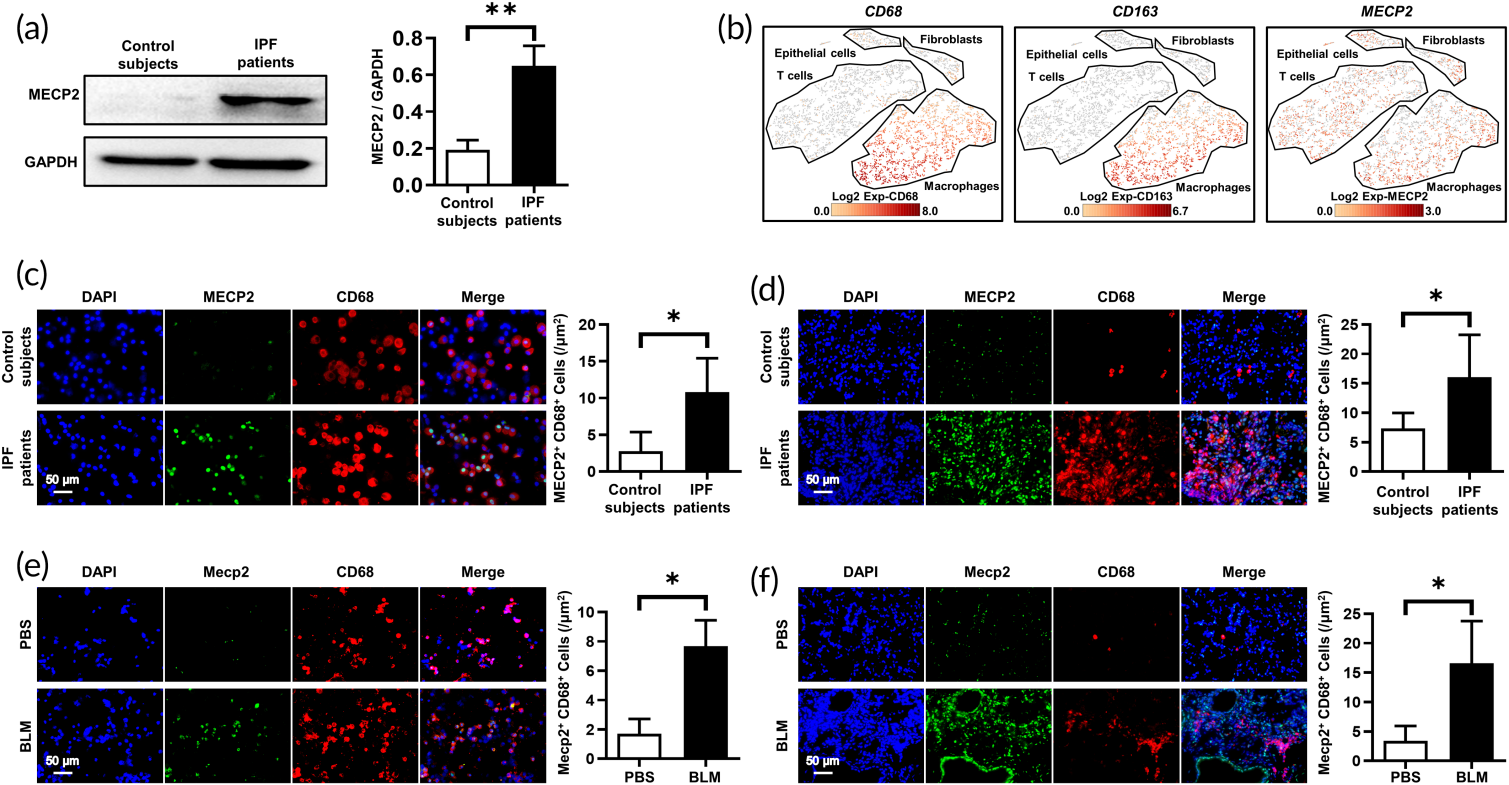
MECP2 expression was increased in lung and BALF samples from IPF patients and mice with pulmonary fibrosis. (a) Western blotting analysis of MECP2 expression in the lung homogenates derived from IPF patients and control subjects. Left panel: Representative Western blotting images. Right panel: A bar graph showing the mean data of all subjects analyzed in each group. (b) Representative images for single‐cell sequencing from two IPF patients. (c and d) Representative results of coimmunostaining for MECP2 and CD68 in lung sections (c) and BALF (d) from IPF patients and control subjects. A total of five patients with IPF and five control subjects were analyzed. (e and f) Representative coimmunostaining results showing Mecp2 and CD68 in BALF (e) and lung sections (f) from BLM‐induced mice with pulmonary fibrosis. The nuclei were stained blue by DAPI, and the images were taken at ×400 magnification. Five mice were included in each study group. *p < 0.05; **p < 0.01; ***p < 0.001. BALF, bronchoalveolar lavage fluid; BLM, bleomycin; IPF, idiopathic pulmonary fibrosis; MECP2, methyl‐CpG‐binding protein 2

To address the role of MECP2 in IPF, we first measured MECP2 expression in the lung tissues of IPF patients and control subjects by Western blotting. Interestingly, significantly high expression of MECP2 was noted in fibrotic lung homogenates (Figure 1a). Next, we sought to identify the cells with altered MECP2 expression. For this purpose, we performed single‐cell sequencing on samples from two IPF patients. Consistently, macrophages (CD68^+^ cells) from IPF patients had an alternatively activated phenotype (CD163^+^ cells) (Figure 1b). Remarkably, MECP2 was highly expressed in macrophages (Figure 1b). To confirm this observation, we conducted immunofluorescent staining of BALF samples from IPF patients and control subjects. Indeed, macrophages were the predominant cell type with MECP2 overexpression, as evidenced by colocalization with CD68^+^ cells (Figure 1c, red). We further examined the lung sections of IPF patients and noted that MECP2 was almost undetectable in the lung sections of control subjects, while IPF patient‐derived lung sections were characterized by macrophage infiltration and MECP2 overexpression (Figure 1d).

To further verify these findings, pulmonary fibrosis model mice were subjected to intratracheal injection of BLM (2 U/kg), and Western blotting was performed in the lung homogenates. Indeed, compared with the PBS treated mice, higher level of Mecp2 was noted in BLM‐induced mice in a time‐dependent manner (Figure S1). Furthermore, coimmunofluorescence staining was prepared on BALF samples and lung sections from PBS‐ or BLM‐treated mice. Consistent with the IPF patients' data, Mecp2 was highly expressed in macrophages from BLM‐induced mice, as demonstrated by the colocalization of Mecp2 (green) with CD68^+^ cells (red, Figure 1e,f). Collectively, our findings indicate that IPF patients and mice with pulmonary fibrosis exhibit higher MECP2 expression in macrophages in the lungs than those of controls.

### 3.2 Silence of Mecp2 expression blunted the M2 program in macrophages by modulating Irf4 expression

**FIGURE 2:**
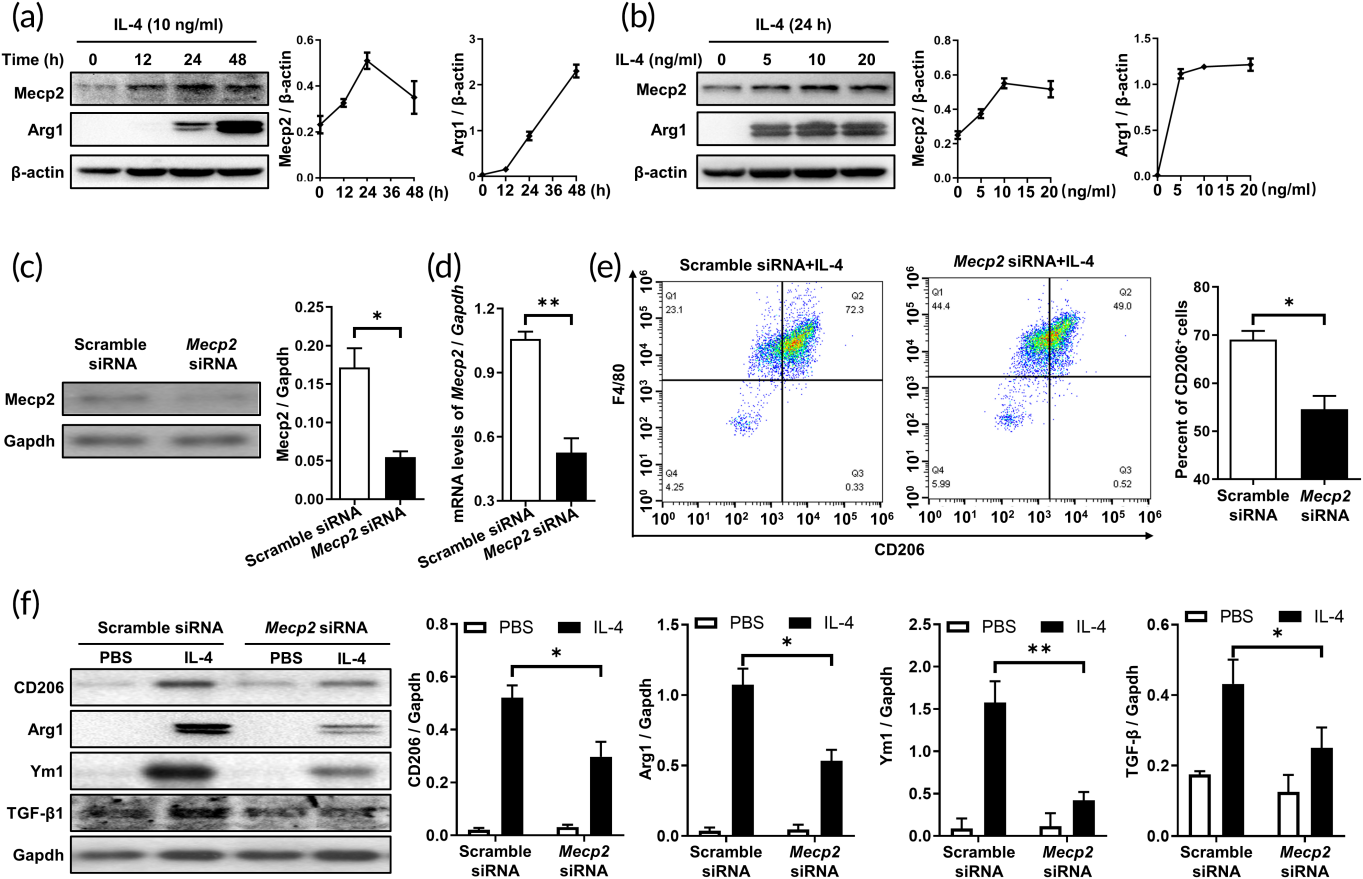
Mecp2 promoted IL‐4‐induced M2 macrophage polarization. (a) Results of time course Western blotting analysis of Mecp2 and Arg1 expression in BMDMs following IL‐4 (10 ng/ml) stimulation. (b) Western blotting analysis of Mecp2 and Arg1 expression in BMDMs following induction with different doses of IL‐4 for 24 h. (c and d) Western blotting (c) and RT‐PCR (d) analyses of the interference efficiency of *Mecp2* siRNA in BMDMs. (e) Flow cytometric analysis of CD206 expression in BMDMs following IL‐4 stimulation. Left panel: Representative flow cytometry results. Right panel: A bar graph showing the data from three replicates. (f) Western blotting analysis of CD206, Ym1, TGF‐β1, and Arg1 expression in BMDMs after IL‐4 stimulation. Left panel: Representative Western blotting images. Right panel: Bar graphs showing the data from three replicates. *p < 0.05; **p < 0.01. Arg1, Arginase 1; BMDMs, bone marrow‐derived macrophages; Mecp2, methyl‐CpG‐binding protein 2; RT‐PCR, reverse transcription‐polymerase chain reaction; siRNA, small interfering RNA; TGF‐β1, transforming growth factor‐beta 1

Next, we conducted Western blotting analysis of BMDMs following IL‐4 stimulation and demonstrated that IL‐4 induced Mecp2 overexpression in a dose‐ and time‐dependent manner during macrophage polarization (Figure 2a,b). These observations reminded us to examine the impact of Mecp2 on the M2 polarization of macrophages. For this purpose, the *Mecp2* siRNA was designed. As expected, Mecp2 was significantly blunted in siRNA‐transfected BMDM after IL‐4 stimulation (Figure 2c). Similar results were also noted by RT‐PCR (Figure 2d). Furthermore, we transfected BMDMs with *Mecp2* siRNA or scramble siRNA (Scr siRNA) before IL‐4 treatment for 24 h. Notably, Mecp2 promoted M2 polarization in macrophages, as illustrated by a lower percentage of F4/80 + CD206+ cells in *Mecp2* siRNA‐transfected BMDMs than in Scr siRNA‐treated BMDMs following IL‐4 stimulation (Figure 2e). In addition, the expressions of CD206, arginase‐1, Ym1, and TGF‐β1, which are produced by M2 macrophages, were attenuated in *Mecp2* siRNA‐treated BMDMs (Figure 2f). In addition, Mecp2 was likely alleviated M1 macrophages activation, as evidenced by lower expression of iNOS was detected in *Mecp2* siRNA‐transfected BMDMs after LPS treatment (Figure S2).

**FIGURE 3:**
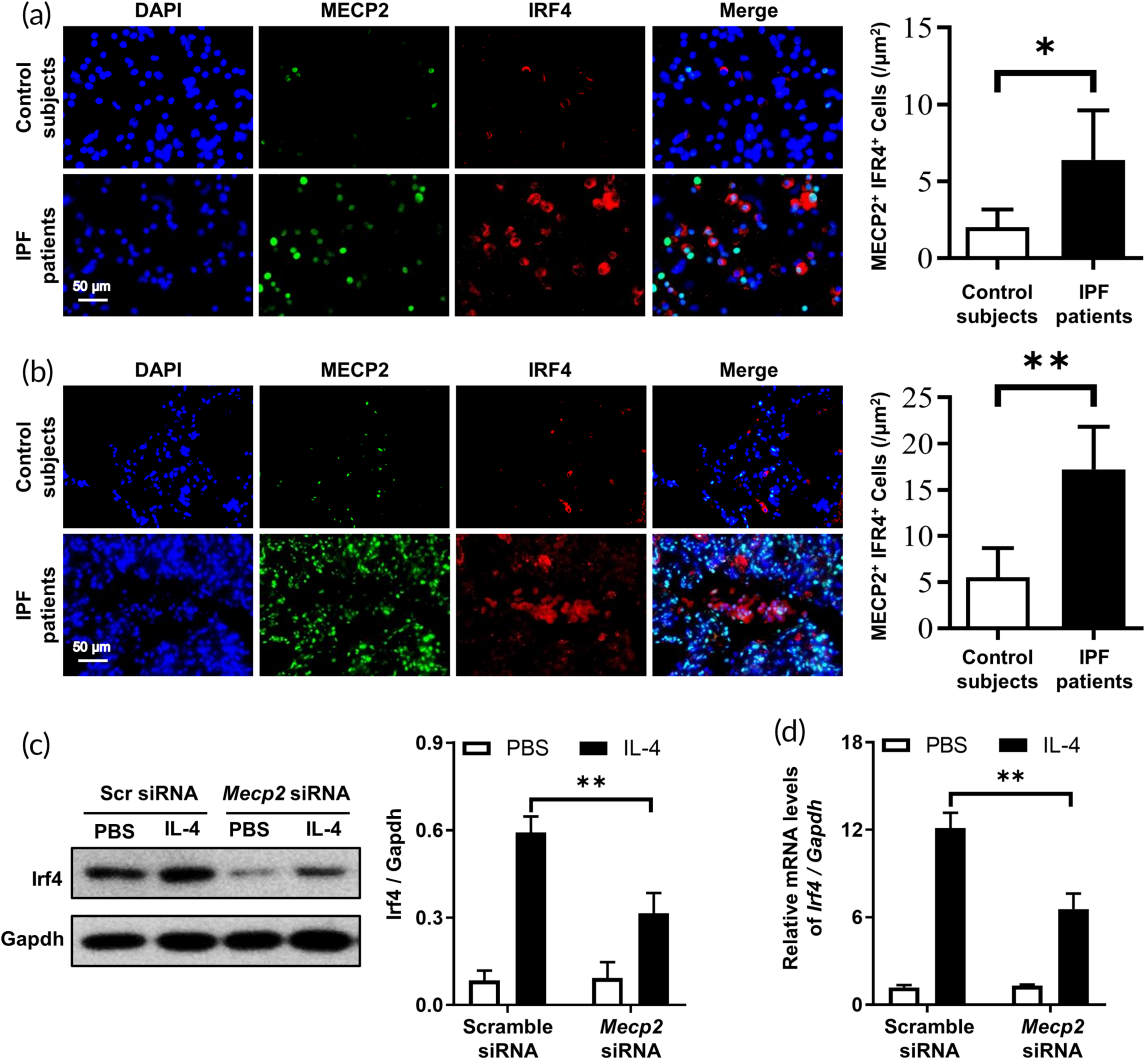
Mecp2 regulates M2 polarization via Irf4. (a and b) Representative images of coimmunostaining for MECP2 and IRF4 in BALF (a) and lung sections (b) from IPF patients and control subjects. The nuclei were stained blue by DAPI, and the images were taken at ×400 magnification. A total of five patients with IPF and five control subjects were analyzed. (c) Western blotting analysis of Irf4 expression in BMDMs after IL‐4 stimulation. Left panel: Representative Western blotting images. Right panel: Bar graphs showing the data from three replicates. (d) RT‐PCR analysis of the expression of *Mecp2* in BMDMs following IL‐4 treatment. *p < 0.05; **p < 0.01. BALF, bronchoalveolar lavage fluid; BMDMs, bone marrow‐derived macrophages; IPF, idiopathic pulmonary fibrosis; IRF4, interferon regulatory factor 4; Mecp2, methyl‐CpG‐binding protein 2; RT‐PCR, reverse transcription‐polymerase chain reaction; Scr: scramble

It is well known that IL‐4/STAT6 signaling is critical for optimal and sustained macrophage M2 polarization upon IL‐4 stimulation.^23^ We then compared changes in the levels of p‐STAT6 between *Mecp2* siRNA‐ or Scr siRNA‐treated BMDMs following IL‐4 stimulation for 1 h. However, no significant difference in p‐STAT6 was detected between *Mecp2* siRNA‐ or Scr siRNA‐treated BMDMs (Figure S3). Given that Mecp2 could repress the transcriptional activity of Pu.1,^24^ and Pu.1 directly binds to the Irf4 promoter and suppresses its expression,^25^ we hypothesized that Mecp2 could exacerbate M2 macrophage polarization by regulating Irf4 expression. Consistent with this hypothesis, the expression of IRF4 (red) was significantly elevated in MECP2‐positive cells (green) in BALF samples and lung sections from IPF patients (Figure 3a,b). Functionally, a marked decrease in Irf4 expression was observed in *Mecp2* siRNA‐transfected BMDMs following 24 h of IL‐4 stimulation (Figure 3c). Consistently, similar results were also observed by RT‐PCR (Figure 3d), indicating that Mecp2 regulated Irf4 in transcriptional level. Collectively, these data show that Mecp2 facilitates M2 macrophage polarization by enhancing Irf4 expression.

### 3.3 Characterization and in vivo biodistribution of *Mecp2* siRNA‐loaded liposomes

**FIGURE 4:**
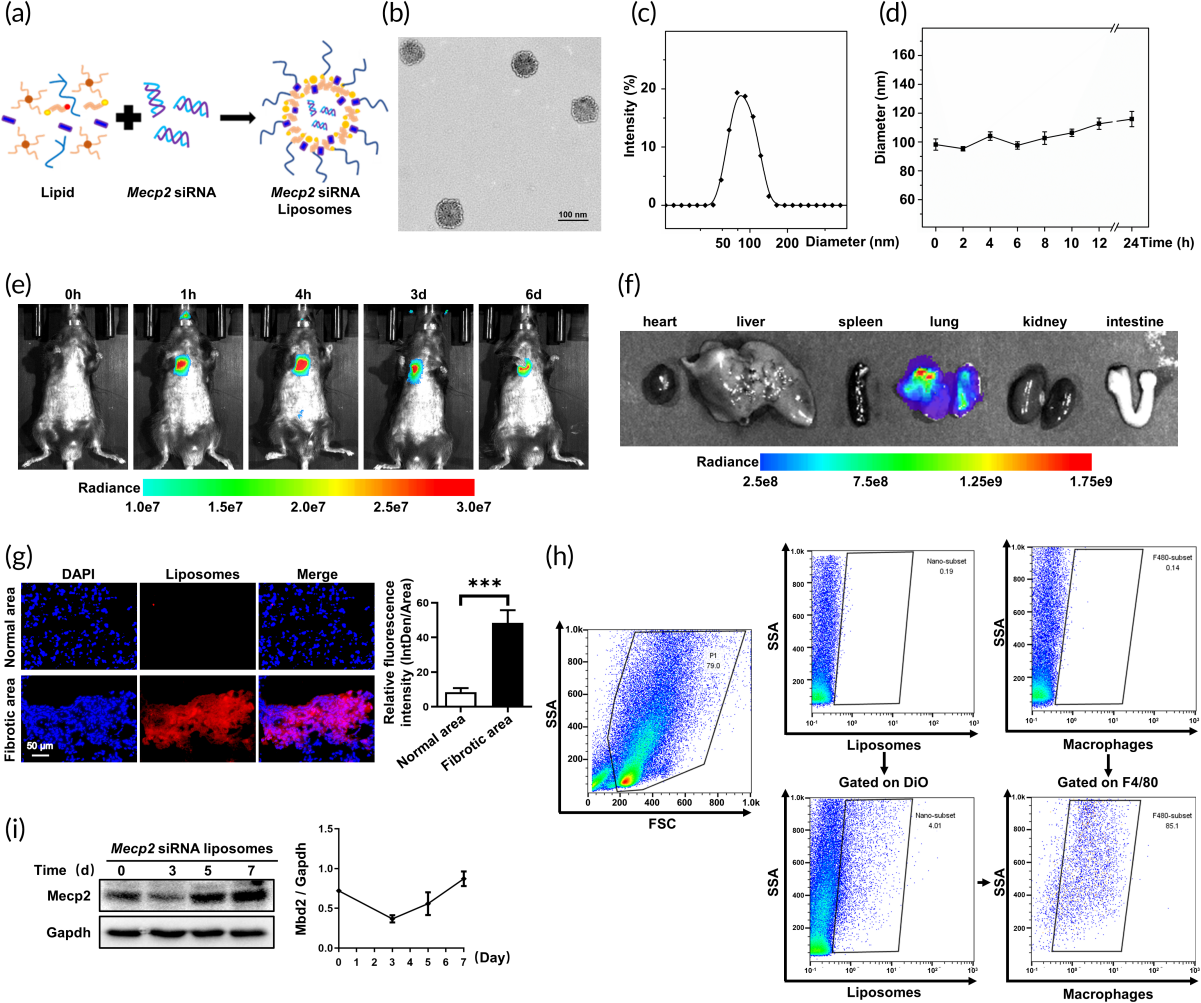
Biodistribution of liposomes after intratracheal injection. (a) Schematic diagram showing the preparation of *Mecp2* siRNA‐loaded liposomes. (b) Representative TEM image of *Mecp2* siRNA‐loaded liposomes. (c) Hydrodynamic diameter distributions of *Mecp2* siRNA‐loaded liposomes. (d) Colloid stability of siRNA‐loaded liposomes in PBS. (e) Representative IVIS images of a mouse at different time points after the administration of DiR‐labeled liposomes. (f) Ex vivo fluorescence images of major organs from mice. (g) Immunofluorescence image showing the biodistribution of DiR‐labeled liposomes (red) in the lungs of BLM‐induced mice. The nuclei were stained blue by DAPI, and the images were taken at ×400 magnification. (h) Flow cytometric analysis of liposomes distribution in the lungs of BLM‐induced mice. (i) Temporal changes in Mecp2 expression in the lungs of *Mecp2* siRNA‐loaded liposomes transfected mice after 14 days of BLM induction. Five mice were included in each study group. ***p < 0.001. BLM, bleomycin; Mecp2, methyl‐CpG‐binding protein 2; PBS, phosphate‐buffered saline; siRNA, small interfering RNA; TEM, transmission electron microscopy

To translate above observations into clinical practice, we used RNAi‐based therapy to reduce the levels of Mecp2 in the lung. First, liposomes carrying *Mecp2* siRNA were prepared (Figure 4a), and then the characteristics of these liposomes were examined. The average hydrodynamic diameters of blank liposomes and siRNA‐loaded liposomes were 103 ± 9 and 96 ± 4 nm, respectively (Figure S4). The zeta potentials of blank liposomes and siRNA‐loaded liposomes were 23.1 ± 2.8 and 3.4 ± 0.6 mV, exhibiting PDIs of 0.11 ± 0.08 and 0.09 ± 0.04, respectively (Figure S4). In addition, the obtained liposomes were able to encapsulate siRNA with a high entrapment efficiency of over 95% and the loading efficiency was around 19.8 ± 1.7% (Figure S4). Subsequently, we evaluated the morphology of the prepared liposomes by TEM. siRNA‐loaded liposomes demonstrated a core‐shell structure with a well‐defined spheroidal shape (Figure 4b). The light‐colored circles around the liposomes represented the lipid layer and darker core was the hydrophilic part inside liposomes stained by phosphotungstic acid. Importantly, siRNA‐loaded liposomes exhibited a uniform distribution and sustained stability for at least 24 h (Figure 4c,d).

Next, we examined the biodistribution of siRNA‐loaded liposomes in vivo. The mice were analyzed by the IVIS system at different time points (0 h, 1 h, 4 h, 3 days and 6 days) following intratracheal injection of DiR‐labeled liposomes. Remarkably, siRNA‐loaded liposomes predominately accumulated in the lung and gradually decreased over time (Figure 4e). Similar results were obtained in ex vivo images (Figure 4f). To further investigate the distribution of liposomes in the fibrotic lung, we examined lung sections from mice with pulmonary fibrosis after intratracheal injection of DiO‐labeled liposomes. Surprisingly, fluorescence was almost undetectable in the normal areas but was obviously concentrated in the fibrotic regions (Figure 4g). Notably, majority of the liposomes were taken up by macrophages, as illustrated by almost 80% DiO^+^ cells being F4/80^+^ cells (Figure 4h).

To address the silencing efficiency of siRNA‐loaded liposomes on Mecp2 expression, we examined temporal changes of Mecp2 expression in the lungs following liposomes‐based pulmonary delivery at Day 14 after BLM induction. Importantly, a significant decrease in Mecp2 expression was detected after intratracheal injection of *Mecp2* siRNA‐loaded liposomes, and the nadir occurred on Day 3 after administration. However, this expression level returned to baseline on Day 5 (Figure 4i). Given that some kinds of nanoparticles could induce severe alveolar injury, we next examined the toxicity of liposomes in vitro and vivo to address this concern. Interestingly, the liposomes showed favorable biocompatibility which was verified by CCK8 assay (Figure S5a). In addition, intratracheal injection of siRNA‐loaded liposomes seemed to be safe, as no obvious alveolar damage of other organs injury was detected (Figure S5b,c).

### 3.4 *Mecp2* siRNA‐loaded liposomes reversed experimental pulmonary fibrosis

**FIGURE 5:**
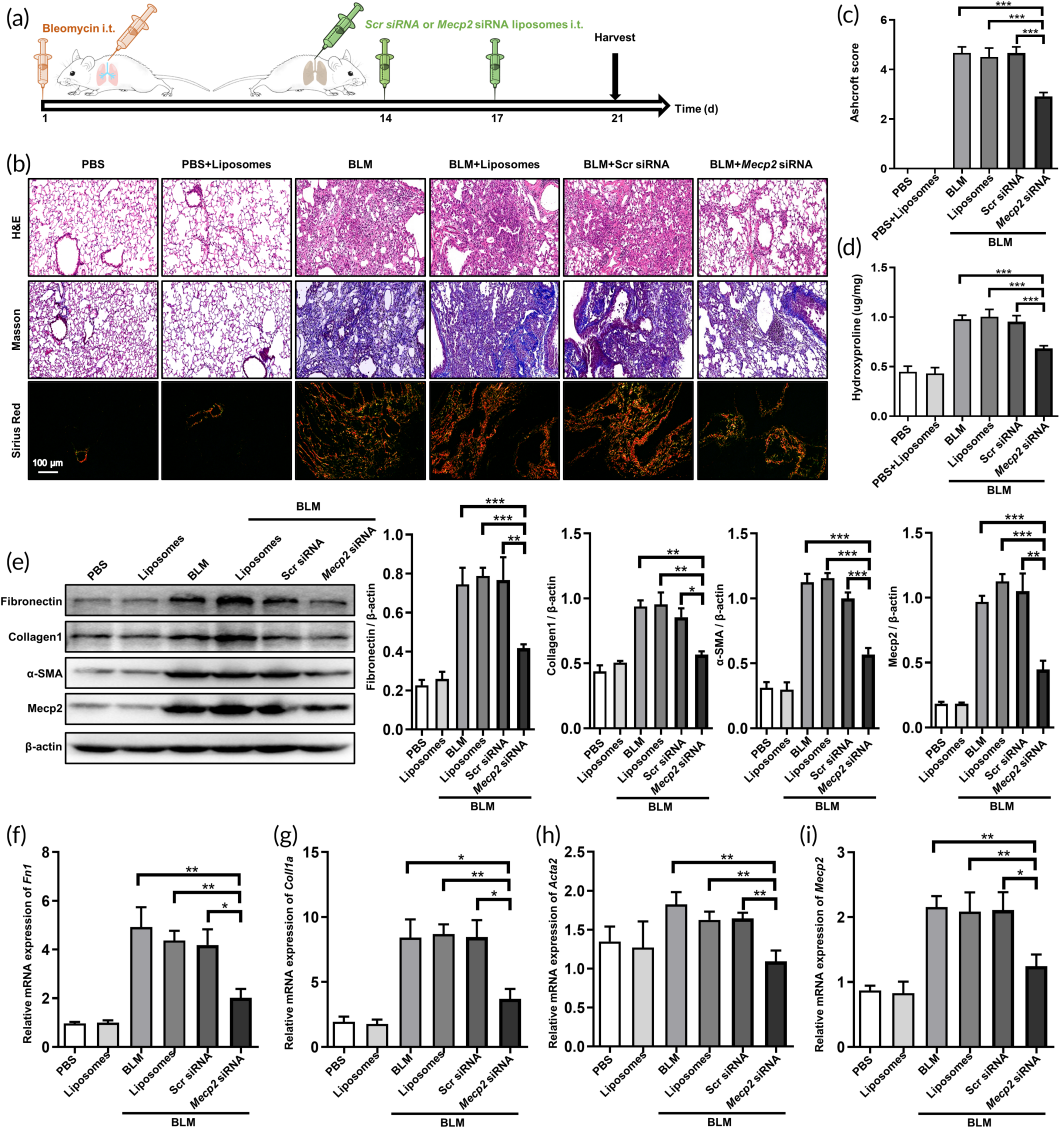
Intratracheal administration of *Mecp2* siRNA‐loaded liposomes reversed established pulmonary fibrosis. (a) Schematic of the experimental design. (b) Histological analysis of the severity of lung fibrosis in mice after BLM induction. Left panel: Representative images of H&E‐ (top), Masson‐ (middle), and Sirius red (bottom)‐stained lung sections. Images were taken at an original magnification of ×200. (c) Ashcroft scores showing the severity of fibrosis. (d) The quantification of hydroxyproline in BLM‐induced mice. (e) Western blotting analysis of fibronectin, collagen 1, α‐SMA, and Mecp2 expression in mice. Left panel: Representative Western blotting images. Right panel: Bar graphs showing the mean data from all subjects analyzed in each group. (f–i) RT‐PCR analysis of *Fn1* (f), *Col1a1* (j), *Acta2* (h), and *Mecp2* (i) expression in each group. Five mice were included in each study group. *p < 0.05; **p < 0.01; ***p < 0.001. BLM, bleomycin; H&E, hematoxylin and eosin; Mecp2, methyl‐CpG‐binding protein 2; RT‐PCR, reverse transcription‐polymerase chain reaction; siRNA, small interfering RNA

Based on the above observation, we sought to assess the curative effect of *Mecp2* siRNA‐loaded liposomes in a model of experimental pulmonary fibrosis. Mice were intratracheally injected with BLM on Day 1, and *Mecp2* siRNA‐loaded liposomes (1 mg/kg) were intratracheally administered on Day 14 and 17. Finally, lung injury and fibrosis were assessed on Day 21 (Figure 5a). Histologically, mice in the BLM, BLM + liposomes and BLM + Scr siRNA‐loaded liposomes groups exhibited severe lung injury (H&E staining) and aberrant collagen accumulation (Masson and Sirius red staining) (Figure 5b). However, drastic improvements in lung injury and fibrosis were observed in *Mecp2* siRNA‐loaded liposomes‐treated mice (Figure 5b). In addition, the severity of pulmonary fibrosis was much lower in the *Mecp2* siRNA‐loaded liposomes‐treated group than in the other groups, as shown by the Ashcroft scores (Figure 5c). The level of hydroxyproline, a major component of all types of fibrillar collagen, was next measured in the lung homogenates. Indeed, the levels of hydroxyproline were significantly diminished in the lungs of *Mecp2* siRNA‐loaded liposomes‐treated mice (Figure 5d).

To further evaluate the therapeutic effects of *Mecp2* siRNA‐loaded liposomes on pulmonary fibrosis, we measured the expression of fibrotic markers (fibronectin, collagen 1, and α‐SMA) in the lung. Indeed, significantly lower expression of fibronectin, collagen 1 and α‐SMA was observed in *Mecp2* siRNA‐loaded liposomes‐treated mice than in other mice (Figure 5e). Consistently, the suppression of Mecp2 in the lung by *Mecp2* siRNA‐loaded liposomes was verified. In addition, similar effects were observed by RT‐PCR (Figure 5f–i).

### 3.5 Knockdown of Mecp2 expression attenuated M2 macrophage polarization in vivo

**FIGURE 6:**
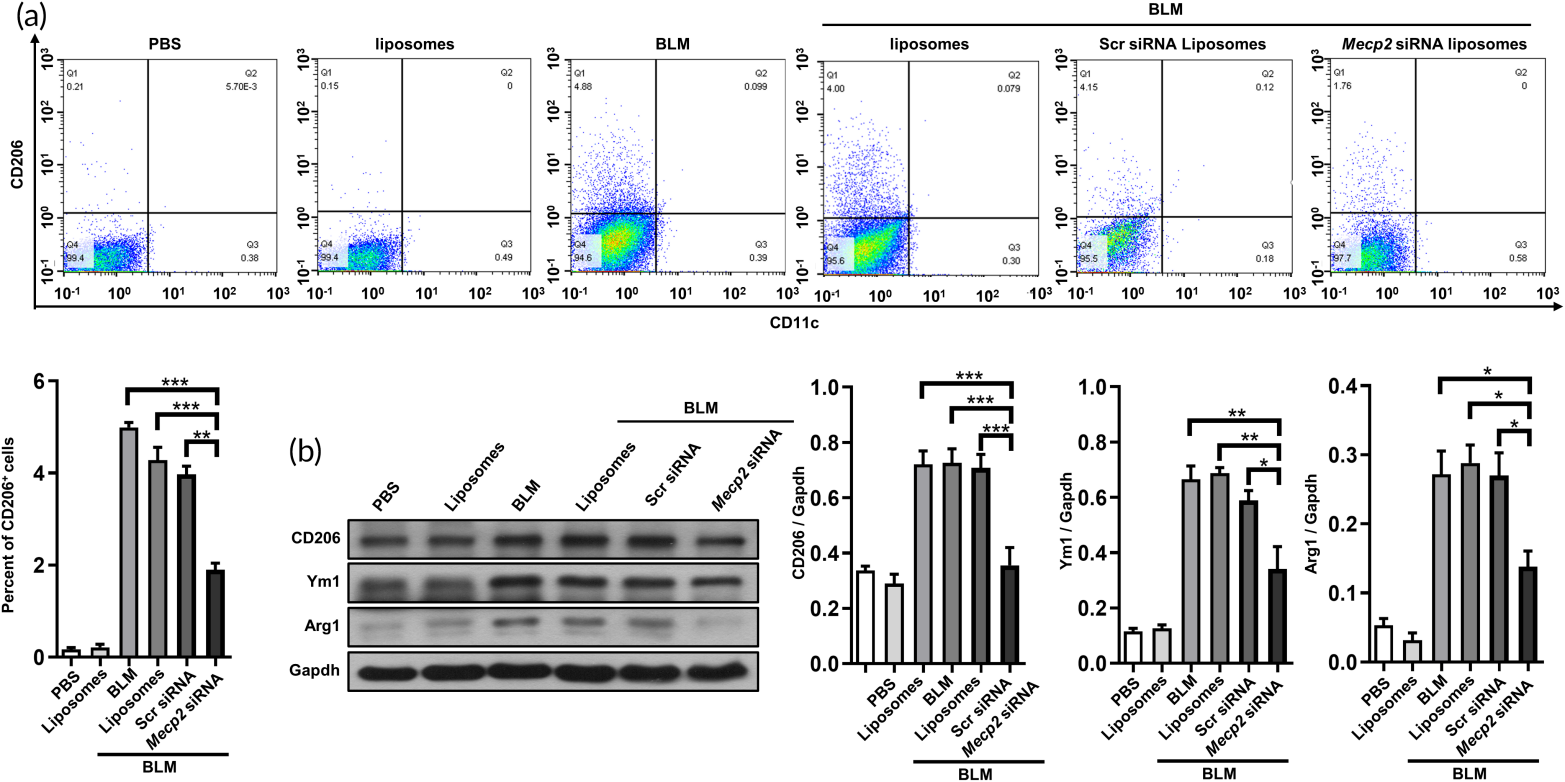
Mecp2 enhanced M2 polarization in BLM‐induced mice. (a) Flow cytometric analysis of M2 macrophages in the lung from each group. Upper panel: Scatter diagrams showing the flow cytometric analysis. Lower panel: A bar graph showing the data. CD206 and CD11c cells were gated from the F4/80^+^ cells. (b) Western blotting analysis of CD206, Ym1 and Arg1 expression in mice. Left panel: Representative Western blotting images. Right panel: Bar graphs showing the data from five mice. *p < 0.05; **p < 0.01; ***p < 0.001. Arg1, Arginase 1; BLM, bleomycin; Mecp2, methyl‐CpG‐binding protein 2; Ym1, chitinase 3‐like 3

To validate the role of Mecp2 in M2 macrophage polarization in vivo, we analyzed the lungs of the abovementioned groups by flow cytometry. Macrophages were gated as the F4/80^+^ population. Indeed, the lungs of *Mecp2* siRNA‐loaded liposomes‐treated mice exhibited markedly lower percentages of M2 macrophages (F4/80^+^CD206^+^CD11c^−^) than those of Scr siRNA‐loaded liposomes‐treated mice (Figure 6a). In addition, the expression of M2 markers (Arg1, CD206, and Ym1) was markedly elevated in BLM‐treated mice, BLM + liposomes‐treated mice and BLM + Scr siRNA‐loaded liposomes‐treated mice, whereas in *Mecp2* siRNA‐loaded liposomes‐treated mice, a substantial reduction in M2 markers was detected (Figure 6b).

Collectively, these data suggest that intratracheal administration of *Mecp2* siRNA‐loaded liposomes protects mice against BLM‐induced lung injury and fibrosis by abrogating M2 macrophage polarization.

### 3.6 Therapeutic effects of *Mecp2* siRNA‐loaded liposomes on pulmonary fibrosis were depend on macrophage

**FIGURE 7:**
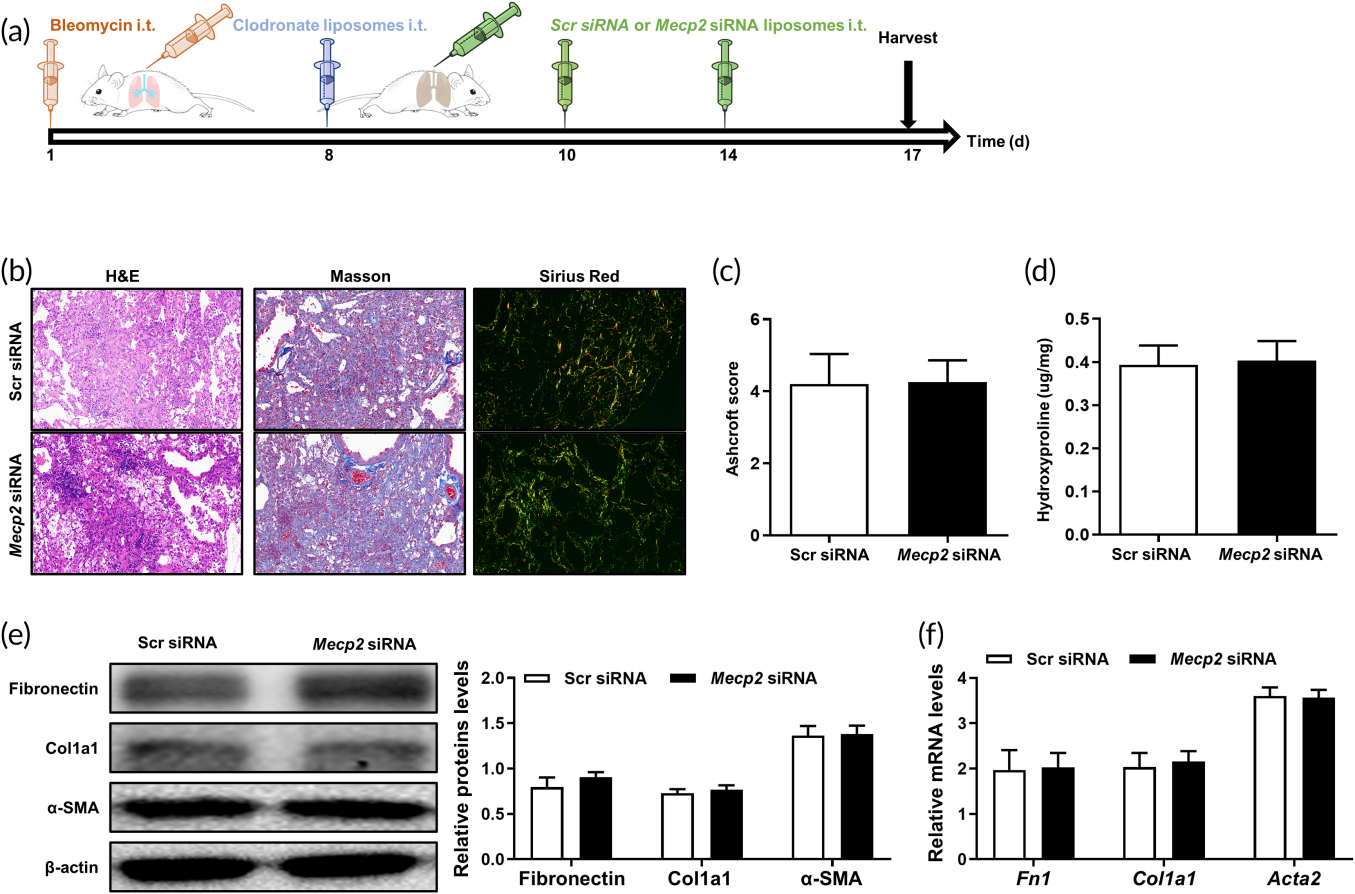
Therapeutic effect of *Mecp2* siRNA‐loaded liposomes on pulmonary fibrosis was depend on macrophages. (a) Schematic of the experimental design. (b) Histological analysis of the severity of lung fibrosis in mice after BLM induction. Left panel: Representative images of H&E‐ (left), Masson‐ (middle), and Sirius red (right)‐stained lung sections. Images were taken at an original magnification of ×200. (c) Ashcroft scores showing the severity of fibrosis. (d) The quantification of hydroxyproline in BLM‐induced mice. (e) Western blotting analysis of fibronectin, collagen 1, α‐SMA, and Mecp2 expression in mice. Left panel: Representative Western blotting images. Right panel: Bar graph showing the mean data from all subjects analyzed in each group. (f) RT‐PCR analysis of *Fn1*, *Col1a1*, and *Acta2* expression in each group. Four mice were included in each study group. BLM, bleomycin; H&E, hematoxylin and eosin; Mecp2, methyl‐CpG‐binding protein 2; RT‐PCR, reverse transcription‐polymerase chain reaction; siRNA, small interfering RNA

The above data prompted us to assume that *Mecp2* siRNA‐loaded liposomes protected mice against BLM‐induced lung fibrosis depending on the repression of macrophage. To address this hypothesis, we first depleted lung macrophages by intratracheal injection of clodronate liposomes and then treated these mice with Scr siRNA‐loaded liposomes or *Mecp2* siRNA‐loaded liposomes (Figure 7a). Finally, the disease severity was assessed on Day 17 after BLM injection. Interestingly, the *Mecp2* siRNA‐loaded liposomes‐treated mice illustrated comparable disease severity as that of Scr siRNA‐loaded liposomes‐treated mice, as demonstrated by pathological staining (Figure 7b,c) and fibrotic indicators (Figure 7d–f), suggesting that the protective effect of Mecp2 knockdown is dependent on macrophages.

## 4 DISCUSSION

**FIGURE 8:**
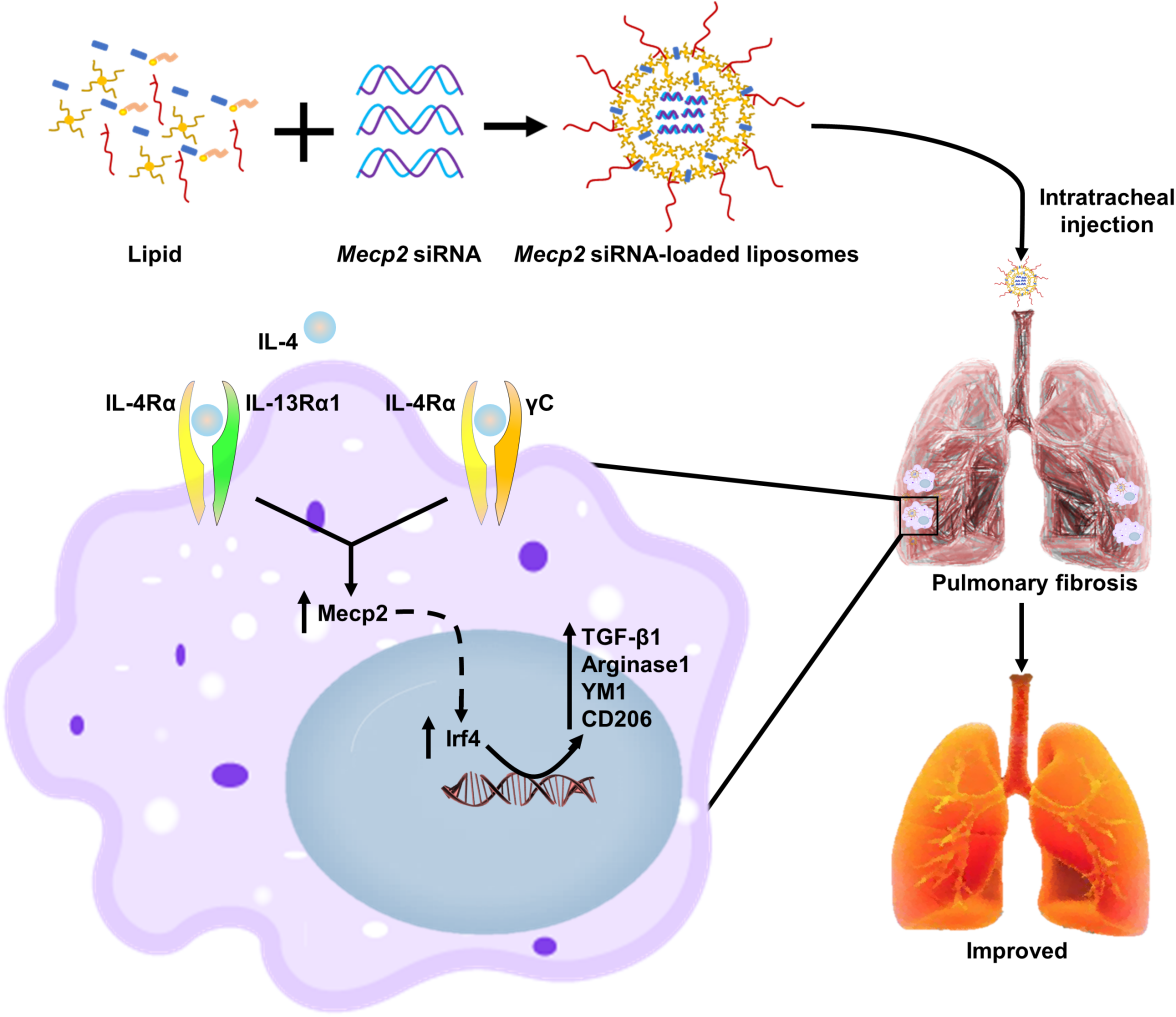
Graphical abstract: Schematic illustration of the protective mechanisms of silencing *Mecp2* in BLM‐induced pulmonary fibrosis through attenuating the macrophage M2 program

IPF is a progressive interstitial lung disease with poor survival and limited treatment options.^1^ Currently, pirfenidone and nintedanib are the only FDA‐approved treatments.^1^, ^26^ However, these drugs do not improve patient outcome and cause frequent adverse events.^26^ Therefore, developing a safe and effective treatment for IPF is urgently needed. In the present study, we showed that MECP2 was upregulated in lung tissues and BALF samples of IPF patients. Specifically, macrophages were one of the main cell types with overexpression of MECP2. In addition, silencing *Mecp2* expression significantly attenuated M2 macrophage polarization by orchestrating Irf4 function. Notably, knockdown of Mecp2 expression in the lung via intratracheal injection of liposomes carrying *Mecp2* siRNA reversed established BLM‐induced lung fibrosis and decreased the infiltration of M2 macrophages in the lung without any side‐effects (Figure 8). Collectively, these results not only highlight the role of DNA methylation in the pathogenesis of IPF but also provide a potent therapeutic strategy for IPF treatment in clinical settings.

DNA methylation is an epigenetic mechanism involving the addition of a methyl group from *S*‐adenosyl methionine to adenine or cytosine to form *N* ^6^‐methyladenine, 5‐methylcytosine, and *N* ^4^‐methylcytosine.^27^ Generally, DNA methylation in the promoter region of a gene represses its transcription. There is compelling evidence that DNA methylation is involved in the pathogenesis of IPF.^28^ Notedly, Mecp2, a member of the MBD protein family which are readers for DNA methylation information, could inhibit or activate gene expression by binding to the CpG island within the gene promoter region. Specifically, Mecp2 acts as a transcriptional repressor when it interacts with 5‐methylcytosine elements at CpG dinucleotides.^29^ In contrast, Mecp2 serves as a transcriptional activator when it binds to 5‐hydroxymethylcytosine.^30^ A previous study demonstrated that Mecp2 may promote the differentiation of fibroblasts to myofibroblasts.^12^ However, the function of Mecp2 in fibrosis is complex, as Mecp2 also exerted antifibrotic effects on dermal fibroblasts from patients with diffuse cutaneous scleroderma.^31^ Surprisingly, other than fibroblasts, macrophages in the lung tissues and BALF samples of IPF patients were characterized by MECP2 overexpression. In addition, stably increased expression of Mecp2 was observed in macrophages following IL‐4 stimulation. In our previous study,^13^ macrophages were characterized by DNA methylation turnover during M2 polarization. Therefore, we examined the effects of Mecp2 on macrophages.

It has been well‐recognized that aberrant macrophage polarization plays a causative role in IPF.^2^ Generally, macrophages are polarized to the M1 phenotype after epithelial injury and perform a proinflammatory role.^4^ Then, M2 macrophages are the major phenotype under the effects of IL‐4 and IL‐13, and these cells control inflammation and accelerate lung repair.^4^ Particularly, M2 macrophages generate a myriad of growth factors, including TGF‐β, PDGF, vascular endothelial growth factor, and insulin‐like growth factor 1, which promote fibroblast activation and ECM deposition.^2^ Additionally, Fizz1 produced by M2 macrophages recruits monocyte‐derived macrophages into the fibrotic region and promotes fibrosis progression.^32^ Thus, repressing the polarization of M2 macrophages, promoting M2 macrophage apoptosis, or blocking the recruitment of mononuclear‐derived macrophages could be a viable therapeutic strategy for IPF. Herein, we revealed that Mecp2 facilitated the polarization of M2 macrophages, as evidenced by decreased levels of M2 markers in IL‐4‐treated BMDMs after silencing *Mecp2* expression.

The next key question was to address the underlying mechanism of Mecp2 predisposing to M2 polarization in macrophages. Previous studies have illustrated that Irf4, a transcription factor, is the critical target gene responsible for controlling M2 macrophage polarization.^33^ Interestingly, compared with control subjects, a significantly higher expression of IRF4 in MECP2‐positive cells was observed in the lung sections and BALF cells from IPF patients. These observations prompted us to examine the impact of Mecp2 on the expression of Irf4 during M2 macrophage polarization. In line with a previous report,^34^ robust levels of Irf4 were detected in macrophages following IL‐4 induction. However, knockdown of *Mecp2* expression strikingly abrogated the IL‐4‐induced Irf4. A previous study showed that Mecp2 could bind to Pu.1 via its N‐terminal domain or transcriptional repression domain and repress the transcriptional activity of Pu.1.^24^ As a critical transcription factor for M2 macrophage polarization,^35^ Pu.1 directly binds to the Irf4 promoter and suppresses Irf4 expression.^25^ However, the underlying mechanism by which Mecp2 regulates Irf4 expression still needs further study.

To translate these observations into clinical treatments, we focused on RNAi‐based therapy.^15^ At present, the main challenge for implementing siRNA therapies into clinical practice is the lack of effective vectors that protect the siRNA against nuclease degradation and deliver it to target cells without severe side‐effects.^36^ Herein, we used a cationic liposome, which was simply constructed, to overcome the abovementioned challenges. Notably, the liposomes carrying siRNA were mainly distributed in the fibrotic area, and 80% were taken up by macrophages after intratracheal administration. Remarkably, *Mecp2* siRNA‐loaded liposomes alleviated the levels of Mecp2 in the lung following BLM induction. Most importantly, intratracheal administration of *Mecp2* siRNA‐loaded liposomes significantly reversed established pulmonary fibrosis and reduced the number of infiltrating M2 macrophages, as shown by the lower number of F4/80^+^CD206^+^CD11c^−^ cells in the lungs than in Scr siRNA‐loaded liposomes‐treated lungs.

## 5 CONCLUSION

In this study, we demonstrate compelling evidence indicating that MECP2 is involved in the pathogenesis of IPF by enhancing M2 macrophage polarization via regulation of IRF4 expression. In addition, intratracheal administration of *Mecp2* siRNA‐loaded liposomes could specifically target macrophages, distribute these liposomes in the fibrotic region and reverse established pulmonary fibrosis without any side‐effects. Taken together, our data show that targeting Mecp2 with siRNA‐loaded liposomes may be a promising strategy for the treatment of pulmonary fibrosis in clinical settings.

## AUTHOR CONTRIBUTIONS

**Yi Wang:** Formal analysis (lead); funding acquisition (lead). **Yong Mou:** Data curation (equal); methodology (equal). **Guo‐Rao Wu:** Data curation (equal); methodology (equal); software (lead). **Qi Wang:** Validation (equal); visualization (lead). **Ting Pan:** Conceptualization (equal); visualization (equal). **Yongjian Xu:** Resources (equal). **Weining Xiong:** Resources (equal). **Qing Zhou:** Writing – original draft (equal); writing – review and editing (equal). **Lei Zhang:** Methodology (equal); software (equal).

## Supporting information


**Figure S1** Western blotting analysis of Mecp2 levels in lung homogenates at different time points after BLM injection. Three mice were included in each study group. BLM: Bleomycin; Mecp2: Methyl‐CpG‐binding protein 2.

**Figure S2** Western blotting analysis of iNOS levels in Scr or *Mecp2* siRNA transduced BMDMs after LPS stimulation. Left panel: Representative Western blotting images. Right panel: bar graphs showing the data with 3 replications. ***, p < 0.001. iNOS: inducible nitric oxide synthase.

**Figure S3** Western blotting analysis of p‐STAT6 levels in Scr or *Mecp2* siRNA treated BMDMs after IL‐4 stimulation for 1 h. Left panel: Representative Western blotting images. Right panel: Bar graphs showing the data with 3 replications. STAT6: Signal transducer and activator of transcription 6.

**Figure S4** The characterization of siRNA‐loaded liposomes.

**Figure S5** The safety of liposomes *in vitro* and *in vivo*. **(a):** The biocompatibility of siRNA‐loaded liposomes to macrophages was evaluated by CCK8 assay. **(b):** Representative images of H&E staining for lung, heart, liver, spleen, kidney and intestine. Images were taken at an original magnification of ×200. **(c):** Liver, cardiac and renal function of mice (n = 5) after siRNA‐loaded liposomes injection. The data are represented as the mean ± SEM. ALT: Alanine aminotransferase; AST: Aspartate aminotransferase; CK: Creatine Kinase; BUN: Blood Urea Nitrogen; CR: Creatinine.

## Data Availability

The data that support the findings of this study are available from the corresponding author upon reasonable request.
