# Supplementary figures and images for: Macrophage‐targeted delivery of siRNA to silence Mecp2 gene expression attenuates pulmonary fibrosis
Source: Bioeng Transl Med. 2022 Jan 18;7(2):e10280. doi: 10.1002/btm2.10280 (PMC9115697; doi:10.1002/btm2.10280)

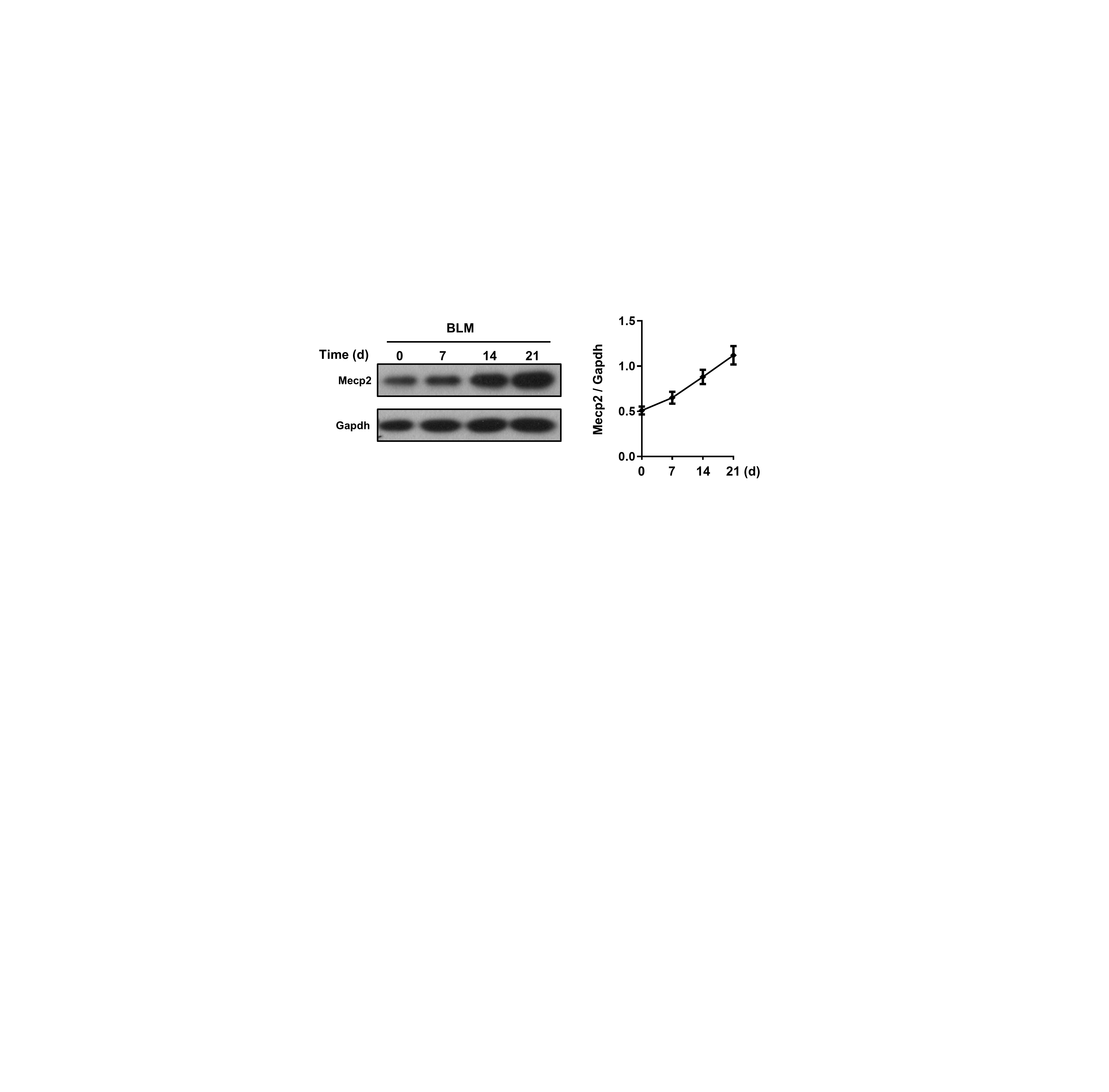

Supplement: Supplementary file 1 — Figure S1 Western blotting analysis of Mecp2 levels in lung homogenates at different time points after BLM injection. Three mice were included in each study group. BLM: Bleomycin; Mecp2: Methyl‐CpG‐binding protein 2. [file BTM2-7-e10280-s004.tif]

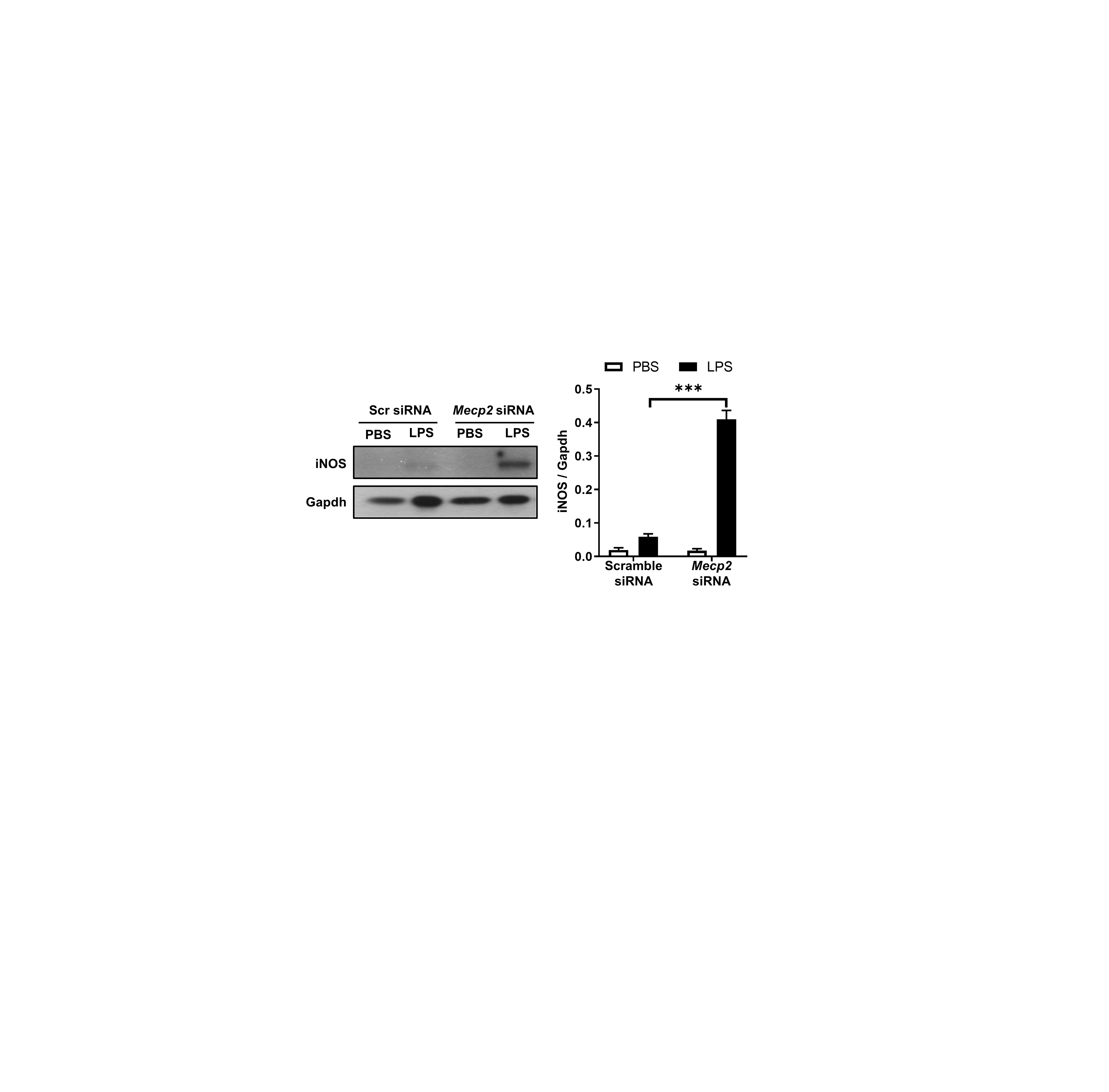

Supplement: Supplementary file 2 — Figure S2 Western blotting analysis of iNOS levels in Scr or Mecp2 siRNA transduced BMDMs after LPS stimulation. Left panel: Representative Western blotting images. Right panel: bar graphs showing the data with 3 replications. ***, p < 0.001. iNOS: inducible nitric oxide synthase. [file BTM2-7-e10280-s003.tif]

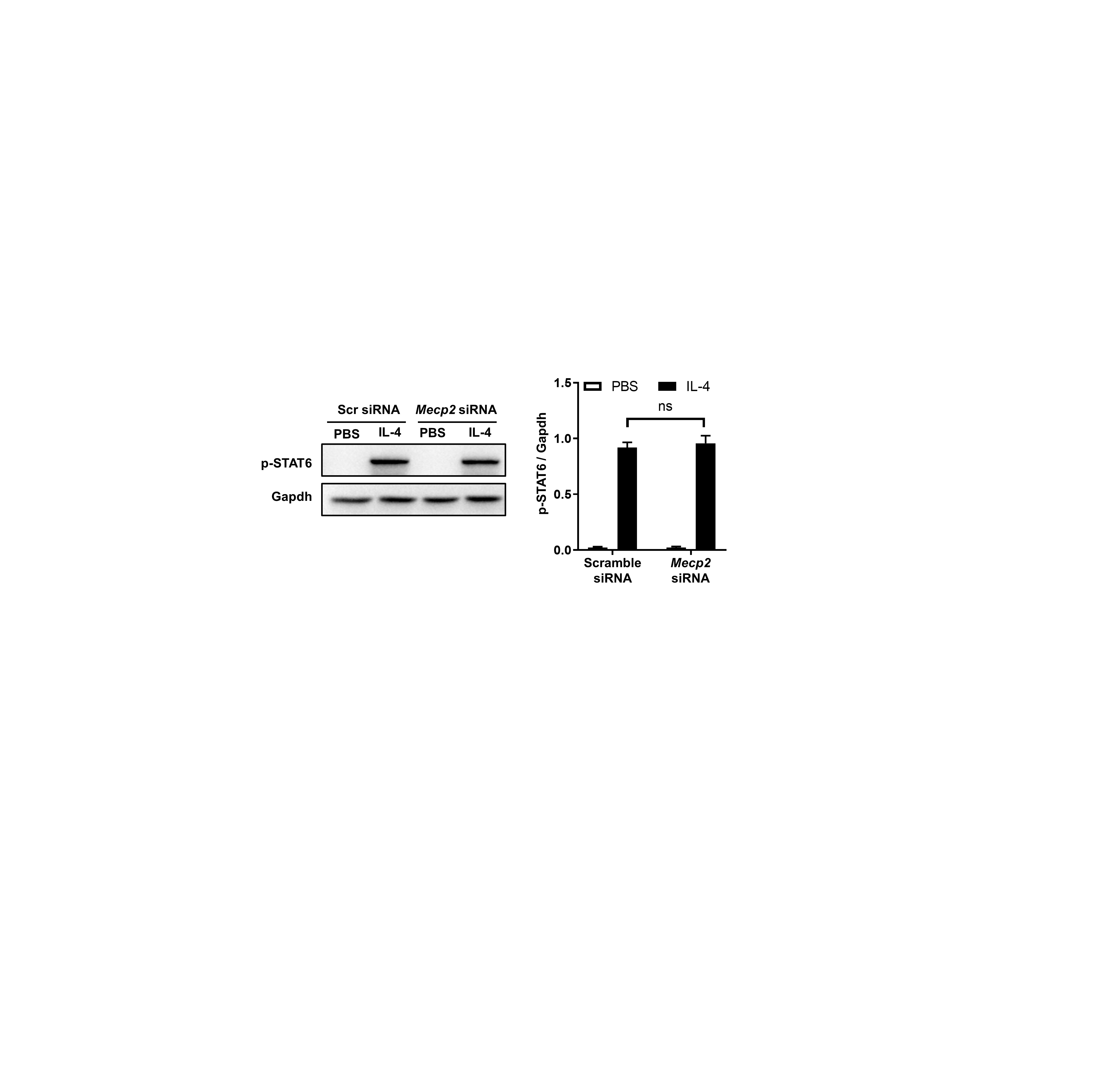

Supplement: Supplementary file 3 — Figure S3 Western blotting analysis of p‐STAT6 levels in Scr or Mecp2 siRNA treated BMDMs after IL‐4 stimulation for 1 h. Left panel: Representative Western blotting images. Right panel: Bar graphs showing the data with 3 replications. STAT6: Signal transducer and activator of transcription 6. [file BTM2-7-e10280-s005.tif]

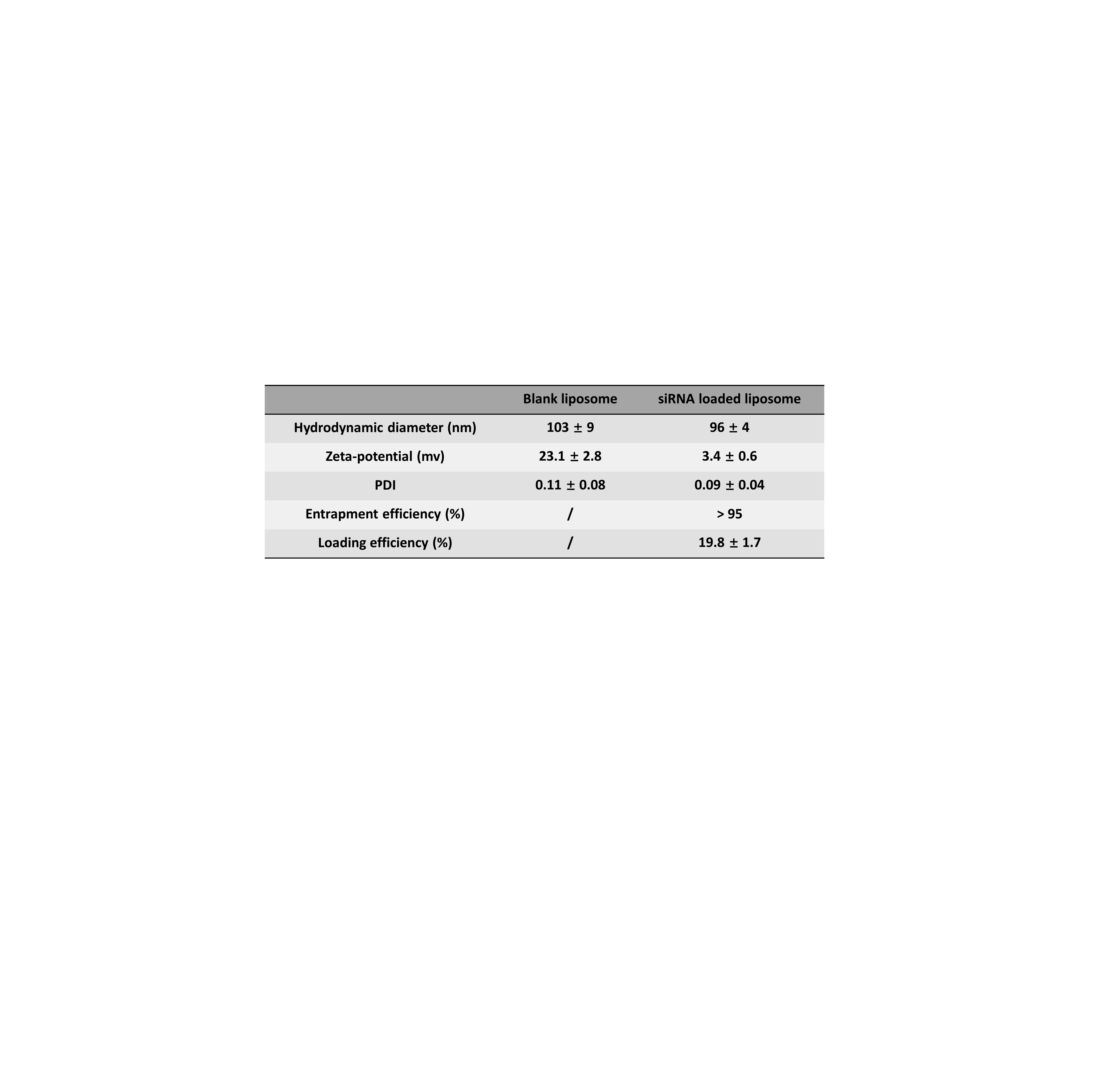

Supplement: Supplementary file 4 — Figure S4 The characterization of siRNA‐loaded liposomes. [file BTM2-7-e10280-s002.tif]

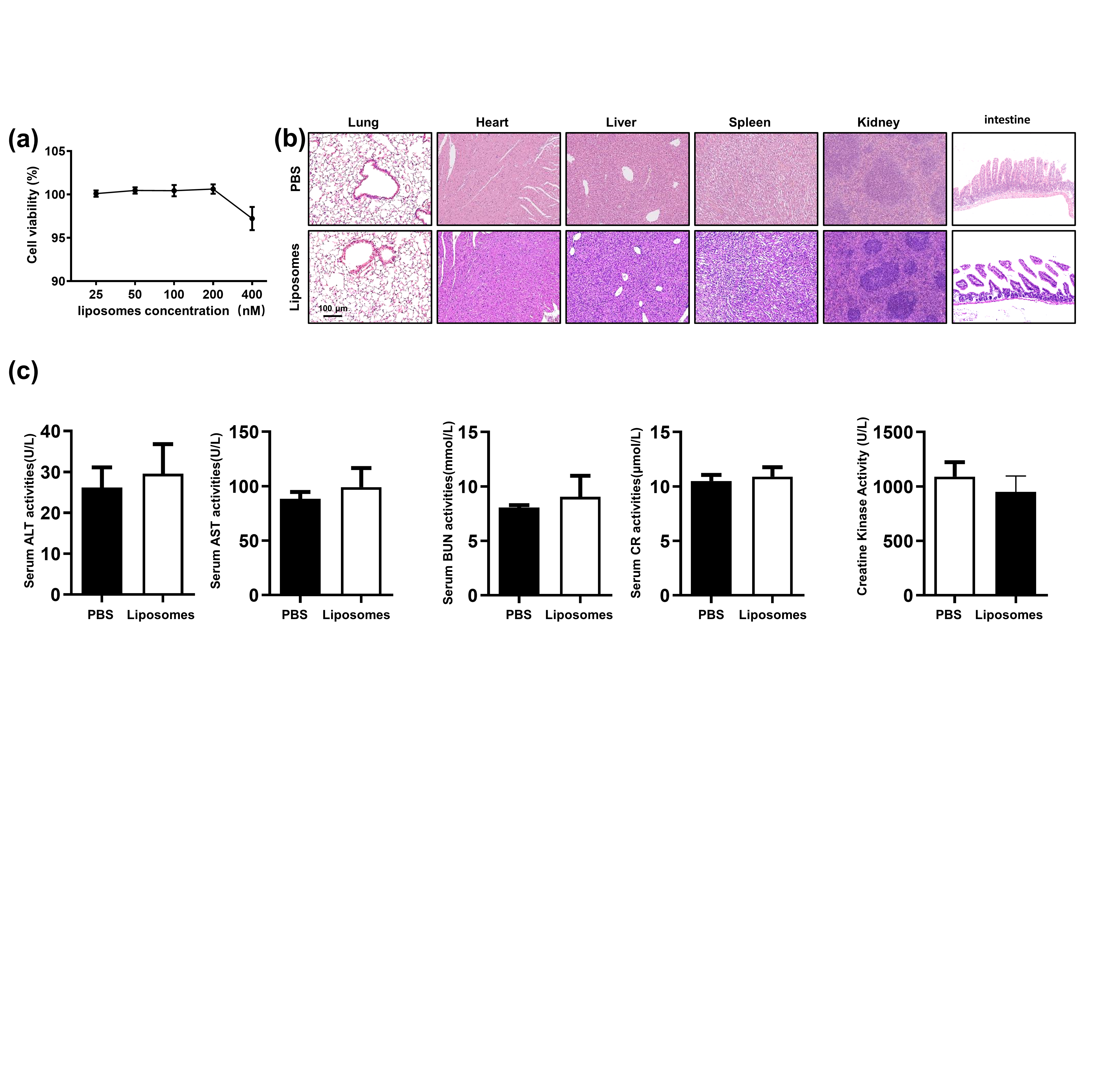

Supplement: Supplementary file 5 — Figure S5 The safety of liposomes in vitro and in vivo. (a): The biocompatibility of siRNA‐loaded liposomes to macrophages was evaluated by CCK8 assay. (b): Representative images of H&E staining for lung, heart, liver, spleen, kidney and intestine. Images were taken at an original magnification of ×200. (c): Liver, cardiac and renal function of mice (n = 5) after siRNA‐loaded liposomes injection. The data are represented as the mean ± SEM. ALT: Alanine aminotransferase; AST: Aspartate aminotransferase; CK: Creatine Kinase; BUN: Blood Urea Nitrogen; CR: Creatinine. [file BTM2-7-e10280-s001.tif]
